# “sCT-Feasibility” - a feasibility study for deep learning-based MRI-only brain radiotherapy

**DOI:** 10.1186/s13014-024-02428-3

**Published:** 2024-03-08

**Authors:** Johanna Grigo, Juliane Szkitsak, Daniel Höfler, Rainer Fietkau, Florian Putz, Christoph Bert

**Affiliations:** 1 Department of Radiation Oncology, Universitätsklinikum Erlangen, Friedrich-Alexander-Universität Erlangen-Nürnberg (FAU), Universitätsstraße 27, DE- 91054 Erlangen, Germany; 2 Comprehensive Cancer Center Erlangen-EMN (CCC ER-EMN), Erlangen, Germany

**Keywords:** MRI, Radiotherapy, MRI-only workflow, sCT, Synthetic CT, Deep learning, Stereotactic radiotherapy, MRonly, Artificial intelligence

## Abstract

**Background:**

Radiotherapy (RT) is an important treatment modality for patients with brain malignancies. Traditionally, computed tomography (CT) images are used for RT treatment planning whereas magnetic resonance imaging (MRI) images are used for tumor delineation. Therefore, MRI and CT need to be registered, which is an error prone process. The purpose of this clinical study is to investigate the clinical feasibility of a deep learning-based MRI-only workflow for brain radiotherapy, that eliminates the registration uncertainty through calculation of a synthetic CT (sCT) from MRI data.

**Methods:**

A total of 54 patients with an indication for radiation treatment of the brain and stereotactic mask immobilization will be recruited. All study patients will receive standard therapy and imaging including both CT and MRI. All patients will receive dedicated RT-MRI scans in treatment position. An sCT will be reconstructed from an acquired MRI DIXON-sequence using a commercially available deep learning solution on which subsequent radiotherapy planning will be performed. Through multiple quality assurance (QA) measures and reviews during the course of the study, the feasibility of an MRI-only workflow and comparative parameters between sCT and standard CT workflow will be investigated holistically. These QA measures include feasibility and quality of image guidance (IGRT) at the linear accelerator using sCT derived digitally reconstructed radiographs in addition to potential dosimetric deviations between the CT and sCT plan. The aim of this clinical study is to establish a brain MRI-only workflow as well as to identify risks and QA mechanisms to ensure a safe integration of deep learning-based sCT into radiotherapy planning and delivery.

**Discussion:**

Compared to CT, MRI offers a superior soft tissue contrast without additional radiation dose to the patients. However, up to now, even though the dosimetrical equivalence of CT and sCT has been shown in several retrospective studies, MRI-only workflows have still not been widely adopted. The present study aims to determine feasibility and safety of deep learning-based MRI-only radiotherapy in a holistic manner incorporating the whole radiotherapy workflow.

**Trial registration:**

NCT06106997.

## Background

Radiotherapy (RT) is an important treatment modality for patients with malignant diseases of the brain such as primary brain tumors and metastases. A further improvement in the local effectiveness of (stereotactic) radiotherapy is likely beneficial for the quality of life and also prognosis of patients receiving RT. Traditionally computed tomography (CT) images are used for planning while magnetic resonance imaging (MRI) images are used for tumor delineation. Therefore, they need to be registered, which is an error prone process [1, 2]. One approach to avoid the registration uncertainty between MRI and CT is the so-called MRI-only workflow, where synthetic CT images are calculated from the MRI data.

Treatment planning relies on high-resolution CT imaging data, which can be transformed into an electron density distribution to model the interaction of the high-energy treatment beam and the human tissue. In conventional RT treatment planning, CT images have been the primary basis of radiotherapy treatment planning and delivery.

CT scans lack sufficient soft tissue contrast in the brain, making it challenging to achieve adequate segmentation of tumor volumes and organs-at-risk (OARs), such as the optic chiasm, and brainstem. Optimization of treatment parameters and evaluation of dose distribution require high resolution and high contrast imaging for target volume definition. Therefore, especially in stereotactic treatments, where high single doses are applied with high precision in one (stereotactic radiosurgery) or a few (fractionated stereotactic radiotherapy) treatment sessions, an MRI scan is essential. The MRI scan typically consists of multiple sequences and provides sufficient soft tissue contrast to differentiate the target volume from healthy brain tissue.

Currently, to incorporate the segmented volumes into the radiation treatment planning, a rigid registration of the CT- and MRI-image is performed. Based on the registration parameters the segmented structures are propagated onto the CT scan. This registration process is subject to uncertainties, which are quantified to be approximately  2 mm [1] and affect all treatment fractions of the patient systematically. Therefore, the registration uncertainty must be considered in the safety margins around the clinical target volume (CTV), resulting in a correspondingly larger planning target volume (PTV). Meanwhile, the typical safety margin from the CTV to the PTV is in the order of only 1–2 mm. Due to these steep gradients, even small deviations can lead to damage of healthy tissue and marginal miss. Therefore, a high degree of accuracy is required throughout the planning process [3–5].

The uncertainties in registration between CT and MRI can be avoided by adopting a solely MRI-based approach, known as the MRI-only workflow. To eliminate the CT from the RT planning process, synthetic CT images (sCT) must be computed from the MRI data to obtain the Hounsfield Units (HU) or electron density information. Besides avoiding registration associated risk of systematic deviations the MRI-only workflow saves time, since patients only need to be scheduled on one imaging modality. Moreover, radiation in the order of few millisieverts of dose from the additional CT examination can be avoided. The reduced registration uncertainty also leads to nominally reduced CTV-PTV safety margins [6].

To compute the sCT from MRI-images different algorithms have been developed over time. The techniques for sCT calculation [7] include bulk-density approaches [8, 9], Atlas- [10, 11], Voxel- [12, 13] and deep learning-based [14, 15] algorithms. Early approaches used a combination of multiple dedicated MRI sequences, such as DIXON, and UTE sequences to distinguish fat, muscle, bone and air [16–20], leading to total acquisition times of around 12–15 min [21], as they required multiple dedicated MRI sequences. In recent years, artificial intelligence-based algorithms have been successfully developed [16, 17, 22]. Unlike voxel, atlas, or bulk-density-based approaches, these algorithms often require only a single sequence. Short DIXON sequences (3:25 min [22, 23]) serve as a basis for these algorithms, which are also part of clinical standards in routine imaging [22]. Dosimetrically, the sCT reconstructed via artificial intelligence approaches matches very well with CT-based clinical standards, with deviations < 1% of the PTV dose [16, 17, 22].

In addition to dose calculation, the sCT must also be suitable for patient positioning and verification. Therefore, MRI scans must be performed with the patient immobilized using a thermoplastic mask in the treatment position. However, this is often not possible in the diagnostic setup because the mask is not compatible with the head receiver coils used in diagnostics. Additionally, MRI-compatible immobilization aids, such as mask holders, are needed as the carbon-fiber-made holders used in radiotherapy are not MRI-compatible. Many medical device manufacturers offer MRI-compatible products for various mask systems, allowing scans in the mask and treatment positions to be performed with flexible coils, leading to sufficiently good images [17, 22] and fewer motion artifacts due to immobilization [24].

After the approval and verification of the radiation treatment plan, the actual radiation treatment is performed. In each treatment session, the patient, immobilized by a mask, must be precisely aligned with the treatment beam as specified in the imaging and treatment planning process. The adjustment of the patient’s position and rotation is based on rigid correction parameters of the treatment table parameters calculated on kV imaging at the medical linear accelerator, where cone-beam CT (CBCT) or 2D/2D X-ray images are created in the treatment position and registered with the CT scan underlying the treatment plan.

It was shown that sCTs can also serve as a reference for CBCT positioning systems, with differences < 1 mm/° compared to CT as the clinical standard [25], whereas deviations in mask alignment and mask holder can reach up to 3 mm/° at maximum [26]. For radiation therapy in the head region, 2D/2D X-ray systems offer a strong correlation between the brain and the skull bones. They are often used because compared to CBCT they are significantly faster, allow for additional checks after table rotation, and require less imaging dose. MRI-only workflows are also possible for such systems, as demonstrated by Masitho et al. [22].

The implementation of MRI-only workflows should ideally consider the mask holder and the mask itself in the dose calculation, even though they have minimal influence on the dose distribution. For stereotactic prescription concepts with 80% of the reference dose, the homogeneity of the dose distribution in the target volume is secondary, and small deviations caused by the (unmodeled) mask should not have a clinical impact. Since these materials are invisible in MRI and often differ between the MRI and CT versions of the mask holder, calculating the mask system as part of sCT creation is challenging. To account for these materials in the sCT, Masitho et al. [27] proposed the use of MRI-visible silicone rods built into the MRI-compatible mask holder, which serve as the basis for creating a model of the mask holder as HU distribution in the planning system. The mask itself is modeled by expanding the patient-air interface. Masitho et al. showed that with this approach, dose differences of 0.2 ± 1.03% can be achieved in the PTV [27]. Taking into account the absolute dosimetry uncertainty, the dosimetric significance is 2% for ΔD50 and ΔD0.01ccm for PTV and OAR [28, 29].

Despite the studies mentioned above, MRI-only workflows are still not widely adopted. This might be due to the limited availability of MRI scanners in radiotherapy clinics or departments. One other explanation could be that the implementation in daily practice is too complex, leading to a lack of acceptance for such workflows. The commercially available sCT software used in this study (CE-labeled sCT algorithm of syngo.via RT Image Suite (VB60) by Siemens Healthineers GmbH) was evaluated for its accuracy in a retrospective research context with 26 patients [2, 27]. Still, proof of feasibility is missing to establish an MRI-only workflow since the whole treatment process of the patient has to be evaluated.

Therefore, this study will prospectively investigate whether an MRI-only workflow is feasible for the stereotactic treatment of patients with brain lesions. In order to enable a smooth integration of the sCT into the clinical routine, the entire radiotherapy chain must be able to be carried out with the help of the sCT. For this purpose, the patients have to be imaged in a radiotherapy mask in the MRI to ensure the same position of the patients in imaging and radiation. Secondly, a dosimetric equivalence of sCT and CT must be assured to enable dosimetrically correct radiation planning for the patients. In addition, mask holder and the mask itself must be correctly integrated into the sCT, as these structures are not visible in the MRI. Furthermore, the patient must be reliably positioned on the treatment couch at each fraction. For this purpose, it must be possible to rely on digitally reconstructed radiography (DRR) from the sCT in order to check the positioning with the help of non-coplanar X-ray images. During the course of this study these parameters are assessed to examine the feasibility of an MRI-only workflow and to identify potential difficulties and exclusion criteria for patients or treatment indications.

## Methods

### Study design

This study is an open, mono-centric, non-randomized, prospective study to evaluate feasibility of an MRI-only workflow providing accurate treatment planning and patient positioning. Patients eligible for the sCT-Feasibility trial require an indication for brain radiotherapy in either the definitive or postoperative setting and must have no MRI contraindications.

**Fig. 1:**
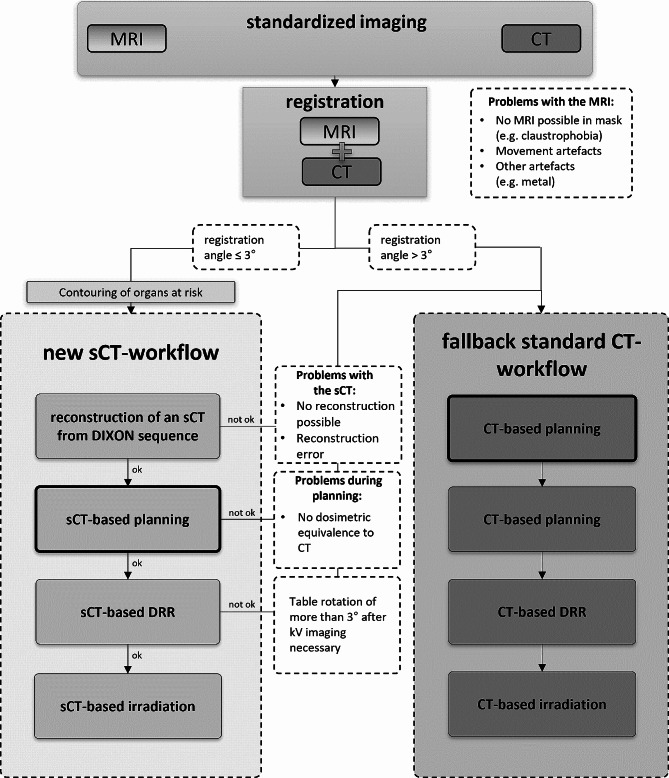
Schematic depiction showing imaging, treatment planning, and radiotherapy of study patients. Each participant of the study will receive MRI and CT scans, as in the standard CT-workflow. The sCT workflow differs only in terms of the data used for treatment planning. The precision and feasibility of the sCT-based process are assessed at various stages during treatment, and if significant deviations occur, the treatment is continued using the conventional clinical standard

Each participant of the study will undergo the standard of care imaging for treatment planning, consisting of both MRI and CT scans, each performed within five days before the commencement of treatment. However, within the study the treatment will be based on MRI-only, i.e. treatment planning and positioning of the patients with 2D/2D imaging at the linear accelerator will be based on synthetic CT data. Feasibility of such a workflow will be evaluated en route by verification against the standard treatment as schematically outlined in Fig. 1 and described in detail in Sect. 2.4.

### Study population

Patients will be informed about the study as part of the initial consultation for radiation therapy.

#### Inclusion criteria

Patients meeting all of the following criteria will be considered for admission to the trial:

- Written informed consent.
- Patient older than 18 years.
- Tumor or metastases in the brain.
- Immobilization with stereotactic mask (basic cranial mask; Brainlab, Munich, Germany).
- Treatment on stereotactic linear accelerator (2.5 mm leafs) equipped with 2D/2D X-ray system.

#### Exclusion criteria

Patients presenting with any of the following criteria will not be included in the trial:

- Metal in the body, metal implants, pacemakers or other patient-specific factors that are a contraindication to an MRI scan.
- Metal implants, pacemakers or other patient-specific factors associated with increased risk from an MRI scan.
- Renal insufficiency (eGFR < 60 ml/min), allergy or other patient-specific factors that constitute a contraindication to contrast administration.
- Renal insufficiency (eGFR < 60 ml/min), allergy or other patient-specific factors associated with an increased risk from contrast administration.
- Patients who are institutionalized in care facilities, prisons or other supervised facilities.
- Patients under guardianship.
- Non-consenting patients.
- Pregnant or breastfeeding patients.

### Study objectives

The main objective of the study is the investigation of the feasibility of an MRI-only workflow using synthetic CT for the treatment of patients with tumors and metastases in the brain. The study will investigate what proportion of treatments can be completed in an MRI-only workflow. The main hypothesis is that 90% of the patients can be successfully treated in an MRI-only workflow, as will be derived in more detail in Sect. 2.5.

The following parameters will be collected as secondary study objectives:

- Which parameters lead to a change from MRI-only workflow to standard workflow?
- Documentation of all comparative parameters between MRI-only and standard workflow.
- Evaluation of the intra-MRI registration data, i.e. comparison of MRI images taken at start vs. end of an MR imaging slot.
- Evaluation of OAR contouring accuracy on MRI data.

### Study plan

During the course of this study the entire treatment procedure is identical for study patients and patients in the standard workflow; it differs only in terms of the data used for treatment planning. As shown in Fig. 1, the accuracy and feasibility of the sCT-based workflow compared to the standard workflow is checked at several points in the treatment process and, in the event of excessive deviations, the treatment is continued according to the clinical standard.

To ensure geometric accuracy of the MRI system a daily system check, including gradient sensitivity, spatial integrity and shimming/ magnetic field homogeneity is performed. Additionally, a monthly check of the gradient non-linearity related distortion, with a dedicated phantom. Also a QA of all coils is regularly performed. The QA program is based on the DEGRO recommendations [30].

The following verifications are investigated, with the detailed parameters/checks listed in Table 1:

**Table 1:** Overview of verifications throughout the treatment workflow. In case a verification fails, the patient is transferred from the MRI-only workflow to the standard workflow based on CT

No.	Verification criterion
1	Can the sCT be generated and is the sCT clinically utilizable?
2	Are the three rotations needed for CT-MRI registration each ≤ 3°?
3	Can a treatment plan be generated and verified using the sCT?
4	Is the dosimetric difference between sCT- and CT-based treatment plans in the PTV ≤ 3%?
5	Is the dosimetric difference between the sCT- and CT-based treatment plans in affected OARs (receiving > 10% of prescribed dose) ≤ 3%?
6	Are the couch correction parameters during patient positioning in the rotational degrees of freedom ≤ 3°?

**Verification 1** aims to ascertain the feasibility of sCT generation and its potential clinical utility. In the course of this study, the patient is subjected to a comprehensive and standardized imaging procedure, encompassing MRI (comprising all sequences of a standard treatment) and CT scans [24, 31]. Subsequently, an sCT is generated exclusively through the reconstruction of MRI data.

As a next step, the process involves registering MRI and CT scans as in the standard workflow in a specialized medical treatment planning system (syngo.via RT Image Suite, VB60a or higher, Siemens Healthineers, Erlangen, Germany). Additionally, the registration process is validated in a second treatment planning system (RayStation v12A or higher, RaySearch, Stockholm, Sweden). **Verification 2** is conducted to assess the degree of rotation during the registration process, i.e. if positioning the patient in the MR scanner was achieved as intended. If all rotation angles are found to be equal to or less than 3°, the workflow remains based on sCT data. However, if both planning systems calculate rotation angles greater than 3°, it is recommended to switch to a CT-based standard workflow. If both registration algorithms calculate different angles, one greater than and one less than 3°, a physician’s clinical manual registration is also used for decision making.

Furthermore, as **additional investigation 1**, the MRI images used for generating the sCT (vibe-DIXON) are registered with the MRI images obtained from the SPACE sequence (utilized for tumor segmentation). The objective is to detect potential changes that might occur during the approximately 30-minute MRI acquisition period, which is specific to this study and does not impact the ongoing treatment.

In the current standard workflow, the clinical target volume (CTV) is contoured on the MRI dataset and transferred to the planning CT. OARs, however, are automatically contoured on the planning CT using the deep learning solution DirectORGANS (Siemens Healthineers GmbH, Erlangen, Germany) that is directly embedded into the CT scanner. In the study, OAR have to be contoured manually based on the MRI data. Retrospectively, as **additional investigation 2**, these OAR structures will be compared with those automatically segmented on CT images (comparison measures include: DICE coefficient and Hausdorff distance).

**Verification 3** is aiming to assess the technical feasibility of treatment planning using the sCT approach, i.e. if the treatment plan can be generated, verified, and transmitted to the record and verify system. The physical treatment planning system used is RayStation (v12A or higher, RaySearch, Stockholm, Sweden). The verification process entails multiple aspects, including the creation of sCT images, consideration of the mask support [27] and verification of the treatment plan (currently Mobius3D, Varian, Darmstadt, Germany and/or ArcCHECK, SunNuclear, Neu-Isenburg, Germany).

For quantitative verification of the sCT-based treatment plan, an equivalent CT-based treatment plan is created by transferring the plan to the CT followed by dose re-calculation using the same physical treatment planning system as mentioned above. The aim of **Verification 4** is to verify the dosimetric equivalence of CT- and sCT-based planning methods. It focuses on assessing the dose difference within the PTV, aiming to ensure that any variations between the CT- and sCT-based plans do not exceed 3% (ΔD_50_ < 3%), where D_50_ refers to the dose covering 50% of the PTV. This rigorous evaluation is crucial to determine the accuracy and consistency of the sCT-based approach in delivering the intended radiation dose to the target volume.

Similarly, **Verification 5** investigates the dose difference in OARs. The objective is to ensure that affected OAR, i.e. OAR which receive > 10% of the prescribed dose, also exhibit dose differences within the acceptable threshold of 3% (i.e. ΔD_50_ < 3% and ΔD_0.01ccm_ <3%). This assessment provides vital insights into the safety and efficacy of the sCT-based radiation planning in sparing at-risk structures from potential damages.

Verification 4 and Verification 5 aim to validate the reliability and clinical suitability of the sCT-based radiation planning method, ensuring that it achieves dosimetric equivalence with the established CT-based approach. This thorough analysis contributes to the overall confidence in implementing sCT-based radiation planning in clinical practice.

As in the standard workflow, patient positioning at the linear accelerator is conducted using the 2D/2D non-coplanar x-ray positioning technique (ExacTrac v6.0.6, Brainlab, Munich, Germany). In case of the MRI-only workflow, the positioning process relies on sCT-based DRRs and thus DRRs reflecting the patient positioning at the MRI acquisition. **Verification 6** focuses on the evaluation of the calculated table rotation for all three directions of rotation. The primary objective is to ensure that the table rotation values do not exceed 3° and that the DRRs calculated from the sCT, can be used correctly. Together with verification 2 this approach serves as a doublecheck that the positioning at the MRI was correct, since positioning of the patient at the MRI, linac and CT should be reproducible and should not differ from each other.

### Statistical design

To estimate the required patient number, the MRI images of all brain stereotactic treatments at the stereotactic linear accelerator of our center, who received a dedicated RT simulation MRI from the last 3 years were examined retrospectively for artifacts and other exclusion criteria of an sCT workflow. The results showed that 91% of the 45 patients would have been suitable for an sCT workflow. 5% of patients were not suitable due to claustrophobia. A further 4% of patients were not eligible because they had metal artifacts on the skull due to surgery or had a glass eye, for example. According to Masitho et al. [25], further exclusion based on dose deviations in OARs or PTVs in comparison to the planning CT are not to be expected. Only one patient was found to have problems with sCT reconstruction. Thus, the overall feasibility of sCTs workflows is estimated to be 90%.

The case number calculation for the study is based on a one-stage design for pilot studies by Fleming [32]. The calculation is based on the following general conditions:

- According to the information of the retrospective analysis of all stereotactic brain patients in the past three years, the application of the sCT workflow is considered feasible if the study procedure described above proves to be feasible accordingly. It is expected and postulated for a positive evaluation of the study outcome that this is the case in 90% of patients.
- The sCT workflow, on the other hand, would be considered not sufficiently feasible if the feasibility rate was 75% or less.
- The probability of erroneously assessing the experimental intervention as feasible even though the true feasibility is at/below 75% (α-error) is said to be only 5%.
- The probability of erroneously discarding the experimental measure as not sufficiently feasible although the true feasibility rate is 90% or more (β-error) should be no more than 10%, corresponding to a power of the study of 90%.

According to these parameters *n* = 54 patients who can be evaluated in terms of feasibility are needed.

Patients who show a violation of the inclusion criteria at the time of inclusion in the study (“non-eligible”) are excluded from the statistical analysis (e.g. Unsuitable for MRI due to metallic implants, withdrawal of consent, immobilization without stereotactic mask). These cases will only be reported casuistically. Which patients with serious protocol violations fall into this category is decided prospectively in a “blind review” before the analyses are carried out.

All other patients are included in the evaluation of the primary objective criterion in the sense of an “intention-to-treat analysis” (ITT).

The primary study objective criterion is calculated by dividing the number of ITT patients in whom the experimental method was found to be feasible by the total number of ITT patients. The exact one-sided 95% and 90% confidence intervals are calculated for the resulting rate.

The analysis of all other parameters is done descriptively, depending on the data type, with frequencies, means, medians, ranges of values and/or confidence intervals, if applicable.

### Risk-benefit assessment

Benefits for participants:

- Optimized target volume definition due to elimination of MRI-CT registration.

Benefit for science and patient treatment:

- Establishment of an MRI-only workflow for radiotherapy of brain tumors or metastases enables radiotherapy planning without MRI-CT registration and the associated potential registration errors.
- Evaluation of sCT-based patient positioning with 2D/2D X-ray system.
- Evaluation of MRI-based contouring of organs at risk compared to CT-based contouring.
- sCT for dose calculation would eliminate the need for an additional conventional CT scan, thereby reducing the radiation dose patients currently receive.

Risks to participants:

- If there are problems during the sCT workflow, treatment might be delayed by half a day for standard CT-based treatment planning to be performed.
- In principle, treatment with sCT can lead to increased dosimetric deviations or the positioning at the linear accelerator may show larger deviations. Since the algorithm used is a medical device, its suitability is given according to the legal requirements. Within the scope of the study a number of verifications against the current clinical standard will be conducted (see Sect. 2.4), so that the risk of increased deviations is further reduced.

### Ethical and legal aspects

The study plan was approved by the Ethics Committee of the Medical Faculty of Friedrich-Alexander-Universität Erlangen-Nürnberg (ID 23-286-Bm). The trial will be carried out by adhering to local legal and regulatory requirements. The protocol will be conducted according to the Guidelines of Good Clinical Practice and the ethical principles described in the Declaration of Helsinki in 2008. This study has been registered prospectively at Clinicaltrails.gov, NCT06106997.

## Discussion

In contrast to CT, MRI enables a superior distinction between cancerous and healthy tissues due to its excellent soft tissue contrast. In current RT planning, CT images are still needed for treatment planning since HUs can be converted to electron density needed for dose calculation. Thus, currently in the clinical workflow MRI and CT images are registered. To avoid this error prone and time-consuming registration process [1, 2], for more than 10 years algorithms for calculation of sCTs from MRI images have been developed and were subsequently tested for dosimetrical equivalence. The existing sCT algorithms are already very reliable [16, 17, 22], but their use in clinical routine is limited. Therefore, the aim of the sCT-feasibility study is to gain and share experience of an MRI-only workflow and establish a successful integration of an MRI-only workflow for brain lesions into clinical routine.

The algorithm used in this study utilizes deep learning to generate the sCT. The accuracy of sCT images produced by deep learning-based techniques relies on the incorporation of relevant image features in the model’s training dataset. Patients treated for brain tumors often have implants or abnormal anatomical structures due to surgery, which can pose a challenge if these anomalies fall outside the scope of the training data. In such cases, the sCT generation software may struggle to interpret the input MR images correctly. To address this issue, it becomes crucial to visually inspect the resulting sCT images to identify potential artifacts. Given these potential inaccuracies in the deep learning reconstruction, the introduction of an MRI-only workflow needs dedicated QA methods [33]. Therefore, in this study, the MRI-only workflow is benchmarked to the standard CT-based workflow during several points during the study (see Table 1). The study aims to identify potential limitations of the patient cohort eligible for an MRI-only workflow. It will also help to identify criteria for a QA of sCT images after the study, i.e. for MRI-only workflows as clinical standard.

In addition to the accurate reconstruction of the sCT, ensuring the correct positioning of the patient in the MRI is essential. Therefore, in this study, the accuracy of patient positioning is assessed at two points based on the registration angle. In future MRI-only workflows, this verification could solely rely on the positioning at the linear accelerator.

Additionally, a suitable and regular QA of the MRI system itself is indispensable to ensure a safe MRI-only workflow [34]. While in the conventional CT-MRI registration-based workflow, artifacts or distortions can be easily identified through the overlap with the geometrically accurate CT, a workflow solely reliant on MRI necessitates a geometrically precise MRI image for accurate treatment planning. This includes assessment of geometric accuracy of the scanner, e.g. gradient nonlinearity and its 3D correction as well as regular QA of the imaging coils and correct adjustment of sequence parameters as e.g. the pixel bandwidth.

This study will be the first clinical prospective feasibility study of MRI-only RT for all brain RT patients. There has been one other feasibility study from Lerner et al. of a deep learning-based MRI-only workflow where 20 out of 21 patients successfully received MRI-only brain RT for gliomas [35]. Similar to our study, MRI Dixon images were used to generate sCT images using a CE-marked deep learning-based software. However, they did exclusively include glioma patients, whereas we are aiming for all brain radiotherapy patients in a larger cohort including stereotactic treatment of brain metastases with mask immobilization.

Our hypothesis is, that 90% of the patients can successfully be treated in an MRI-only workflow. This is in line with the result of Lerner et al. and also published implementation studies on MRI-only RT for prostate cancer treatment with success rates between 87.5 and 100% [36–38].

## Data Availability

Not applicable.

## Abbreviations

CT: Computed Tomography
CBCT: Cone Beam Computed Tomography
CTV: Clinical Target Volume
DVH: Dose Volume Histogram
DRR: Digitally Reconstructed Radiography
HU: Hounsfield Unit
IGRT: Image Guided Radiotherapy
ITT: Intention to Treat
MRI: Magnetic Resonance Imaging
OAR: Organs at Risk
PTV: Planning Target Volume
QA: Quality Assurance
RT: Radiotherapy
sCT: synthetic Computed Tomography

## References

[1] Ulin K, Urie MM, Cherlow JM (2010). Results of a multi-institutional benchmark test for cranial CT/MR image registration. Int J Radiat Oncol Biol Phys.

[2] Masitho S, Putz F, Mengling V, Reißig L, Voigt R, Bäuerle T (2022). Accuracy of MRI-CT registration in brain stereotactic radiotherapy: impact of MRI acquisition setup and registration method. Z Med Phys.

[3] Guckenberger M, Baus WW, Blanck O, Combs SE, Debus J, Engenhart-Cabillic R (2020). Definition and quality requirements for stereotactic radiotherapy: consensus statement from the DEGRO/DGMP Working Group Stereotactic Radiotherapy and Radiosurgery. Strahlenther Onkol.

[4] Seung SK, Larson DA, Galvin JM, Mehta MP, Potters L, Schultz CJ (2013). American College of Radiology (ACR) and American Society for Radiation Oncology (ASTRO) practice Guideline for the performance of stereotactic radiosurgery (SRS). Am J Clin Oncol.

[5] Kocher M, Wittig A, Piroth MD, Treuer H, Seegenschmiedt H, Ruge M (2014). Stereotactic radiosurgery for treatment of brain metastases. A report of the DEGRO Working Group on Stereotactic Radiotherapy. Strahlenther Onkol.

[6] Nyholm T, Nyberg M, Karlsson MG, Karlsson M (2009). Systematisation of spatial uncertainties for comparison between a MR and a CT-based radiotherapy workflow for prostate treatments. Radiat Oncol.

[7] Johnstone E, Wyatt JJ, Henry AM, Short SC, Sebag-Montefiore D, Murray L (2018). Systematic review of Synthetic computed Tomography Generation methodologies for Use in magnetic resonance imaging–only Radiation Therapy. Int J Radiat Oncol Biol Phys.

[8] Wang C, Chao M, Lee L, Xing L (2008). MRI-based treatment planning with electron density information mapped from CT images: a preliminary study. Technol Cancer Res Treat.

[9] Stanescu T, Jans HS, Pervez N, Stavrev P, Fallone BG (2008). A study on the magnetic resonance imaging (MRI)-based radiation treatment planning of intracranial lesions. Phys Med Biol.

[10] Edmund J, Nyholm T (2017). A review of substitute CT generation for MRI-only radiation therapy. Radiat Oncol.

[11] Dowling JA, Lambert J, Parker J, Salvado O, Fripp J, Capp A (2012). An atlas-based electron density mapping method for magnetic resonance imaging (MRI)-alone treatment planning and adaptive MRI-based prostate radiation therapy. Int J Radiat Oncol Biol Phys.

[12] Korhonen J, Kapanen M, Keyriläinen J, Seppälä T, Tenhunen M (2014). A dual model HU conversion from MRI intensity values within and outside of bone segment for MRI-based radiotherapy treatment planning of prostate cancer. Med Phys.

[13] Johansson A, Karlsson M, Yu J, Asklund T, Nyholm T (2012). Voxel-wise uncertainty in CT substitute derived from MRI. Med Phys.

[14] Boulanger M, Nunes J-C, Chourak H, Largent A, Tahri S, Acosta O (2021). Deep learning methods to generate synthetic CT from MRI in radiotherapy: a literature review. Phys Med.

[15] Tang B, Wu F, Fu Y, Wang X, Wang P, Orlandini LC (2021). Dosimetric evaluation of synthetic CT image generated using a neural network for MR-only brain radiotherapy. J Appl Clin Med Phys.

[16] Paradis E, Cao Y, Lawrence TS, Tsien C, Feng M, Vineberg K (2015). Assessing the Dosimetric Accuracy of Magnetic Resonance-Generated Synthetic CT Images for focal Brain VMAT Radiation Therapy. Int J Radiat Oncol Biol Phys.

[17] Dinkla AM, Wolterink JM, Maspero M, Savenije MHF, Verhoeff JJC, Seravalli E (2018). MR-Only Brain Radiation Therapy: dosimetric evaluation of synthetic CTs generated by a dilated convolutional neural network. Int J Radiat Oncol Biol Phys.

[18] Burgos N, Cardoso MJ, Guerreiro F, Veiga C, Modat M, McClelland J et al. Robust CT Synthesis for Radiotherapy Planning: Application to the Head and Neck Region. MICCAI. 2015:476– 84.

[19] Hsu SH, Cao Y, Lawrence TS, Tsien C, Feng M, Grodzki DM (2015). Quantitative characterizations of ultrashort echo (UTE) images for supporting air-bone separation in the head. Phys Med Biol.

[20] Zheng W, Kim JP, Kadbi M, Movsas B, Chetty IJ, Glide-Hurst CK (2015). Magnetic resonance-based Automatic Air Segmentation for Generation of Synthetic Computed Tomography scans in the Head Region. Int J Radiat Oncol Biol Phys.

[21] Hsu S-H, Cao Y, Huang K, Feng M, Balter JM (2013). Investigation of a method for generating synthetic CT models from MRI scans of the head and neck for radiation therapy. Phys Med Biol.

[22] Masitho S, Szkitsak J, Grigo J, Fietkau R, Putz F, Bert C (2022). Feasibility of artificial-intelligence-based synthetic computed tomography in a magnetic resonance-only radiotherapy workflow for brain radiotherapy: two-way dose validation and 2D/2D kV-image-based positioning. Phys Imaging Radiat Oncol.

[23] Hoesl M, Corral NE, Mistry N, MR-based Synthetic (2022). CT reimagined: an AI.based algorithm for continuous hounsfield units in the pelvis and brain - with syngo.via RT Image suite (VB60).

[24] Mengling V, Bert C, Perrin R, Masitho S, Weissmann T, Mansoorian S (2021). Implementation of a dedicated 1.5 T MR scanner for radiotherapy treatment planning featuring a novel high-channel coil setup for brain imaging in treatment position. Strahlenther Onkol.

[25] Liu X, Emami H, Nejad-Davarani SP, Morris E, Schultz L, Dong M (2021). Performance of deep learning synthetic CTs for MR-only brain radiation therapy. J Appl Clin Med Phys.

[26] Barnes M, Yeo A, Thompson K, Phillips C, Kron T, Hardcastle N (2020). A retrospective analysis of setup and intrafraction positional variation in stereotactic radiotherapy treatments. J Appl Clin Med Phys.

[27] Masitho S, Grigo J, Brandt T, Lambrecht U, Szkitsak J, Weiss A (2023). Synthetic CTs for MRI-only brain RT treatment: integration of immobilization systems. Strahlenther Onkol.

[28] Korsholm ME, Waring LW, Edmund JM (2014). A criterion for the reliable use of MRI-only radiotherapy. Radiat Oncol.

[29] Ahnesjö A, Aspradakis MM (1999). Dose calculations for external photon beams in radiotherapy. Phys Med Biol.

[30] Putz F, Bock M, Schmitt D, Bert C, Blanck O, Ruge MI (2024). Quality requirements for MRI simulation in cranial stereotactic radiotherapy: a guideline from the German taskforce imaging in Stereotactic Radiotherapy. Strahlenther Onkol.

[31] Putz F, Mengling V, Perrin R, Masitho S, Weissmann T, Rösch J (2020). Magnetic resonance imaging for brain stereotactic radiotherapy. Strahlenther Onkol.

[32] Fleming TR (1982). One-sample multiple testing procedure for phase II clinical trials. Biometrics.

[33] Vandewinckele L, Claessens M, Dinkla A, Brouwer C, Crijns W, Verellen D (2020). Overview of artificial intelligence-based applications in radiotherapy: recommendations for implementation and quality assurance. Radiother Oncol.

[34] Glide-Hurst CK, Paulson ES, McGee K, Tyagi N, Hu Y, Balter J (2021). Task group 284 report: magnetic resonance imaging simulation in radiotherapy: considerations for clinical implementation, optimization, and quality assurance. Med Phys.

[35] Lerner M, Medin J, Jamtheim Gustafsson C, Alkner S, Olsson LE. Prospective clinical feasibility study for MRI-Only brain Radiotherapy. Front Oncol. 2022;11. 10.3389/fonc.2021.812643. 10.3389/fonc.2021.812643 PMC8784680 35083159

[36] Greer P, Martin J, Sidhom M, Hunter P, Pichler P, Choi JH, et al. A multi-center prospective study for implementation of an MRI-Only prostate Treatment Planning Workflow. Front Oncol. 2019;9. 10.3389/fonc.2019.00826. 10.3389/fonc.2019.00826 PMC6727318 31555587

[37] Tenhunen M, Korhonen J, Kapanen M, Seppälä T, Koivula L, Collan J (2018). MRI-only based radiation therapy of prostate cancer: workflow and early clinical experience. Acta Oncol.

[38] Persson E, Jamtheim Gustafsson C, Ambolt P, Engelholm S, Ceberg S, Bäck S (2020). MR-PROTECT: clinical feasibility of a prostate MRI-only radiotherapy treatment workflow and investigation of acceptance criteria. Radiat Oncol.

